# Nanoparticles in Endodontics Disinfection: State of the Art

**DOI:** 10.3390/pharmaceutics14071519

**Published:** 2022-07-21

**Authors:** Xavier Roig-Soriano, Eliana B. Souto, Firas Elmsmari, Maria Luisa Garcia, Marta Espina, Fernando Duran-Sindreu, Elena Sánchez-López, Jose Antonio González Sánchez

**Affiliations:** 1 Department of Pharmacy, Pharmaceutical Technology and Physical Chemistry, Faculty of Pharmacy and Food Sciences, University of Barcelona, 08028 Barcelona, Spain; xroigsor7@alumnes.ub.edu (X.R.-S.); marisagarcia@ub.edu (M.L.G.); m.espina@ub.edu (M.E.); 2 Department of Pharmaceutical Technology, Faculty of Pharmacy, University of Porto, Rua de Jorge Viterbo Ferreira, 228, 4050-313 Porto, Portugal; 3 REQUIMTE/UCIBIO, Faculty of Pharmacy, University of Porto, Rua de Jorge Viterbo Ferreira, 228, 4050-313 Porto, Portugal; 4 Department of Clinical Sciences, College of Dentistry, Ajman University, University Street Al Jerf 1, Ajman 346, United Arab Emirates; f.elmsmari@ajman.ac.ae; 5 Center of Medical and Bio-Allied Health Sciences Research, Ajman University, University Street Al Jerf 1, Ajman 346, United Arab Emirates; 6 Institute of Nanoscience and Nanotechnology (IN2UB), University of Barcelona, 08028 Barcelona, Spain; 7 Department of Endodontics, Faculty of Dentistry, Universitat Internacional de Catalunya, 08017 Barcelona, Spain; fduran@uic.es (F.D.-S.); jagonzalez@uic.es (J.A.G.S.); 8 Unit of Synthesis and Biomedical Applications of Peptides, IQAC-CSIC, 08034 Barcelona, Spain

**Keywords:** nanoparticles, endodontics, PLGA, metal nanoparticles, dentistry

## Abstract

Endodontic-related diseases constitute the fourth most expensive pathologies in industrialized countries. Specifically, endodontics is the part of dentistry focused on treating disorders of the dental pulp and its consequences. In order to treat these problems, especially endodontic infections, dental barriers and complex root canal anatomy should be overcome. This constitutes an unmet medical need since the rate of successful disinfection with the currently marketed drugs is around 85%. Therefore, nanoparticles constitute a suitable alternative in order to deliver active compounds effectively to the target site, increasing their therapeutic efficacy. Therefore, in the present review, an overview of dental anatomy and the barriers that should be overcome for effective disinfection will be summarized. In addition, the versatility of nanoparticles for drug delivery and their specific uses in dentistry are comprehensively discussed. Finally, the latest findings, potential applications and state of the art nanoparticles with special emphasis on biodegradable nanoparticles used for endodontic disinfection are also reviewed.

## 1. Introduction

The nanotechnological field has highly evolved during the last few decades. Progress achieved in nanotechnology has allowed nanoparticles (NPs) to be considered one of the most promising vehicles in the administration of medicines [1]. Therefore, nanotechnology has been used with highly interesting results in the diagnosis and treatment of several pathologies and also to produce biocompatible materials [2,3,4]. Nevertheless, one of the most relevant purposes of NPs is to act as vehicles to deliver active compounds for imaging and therapeutic agents, such as small molecules, proteins, peptides, and nucleic acids. The primary advantages of NPs are their specificity, low toxicity, targeting and biocompatibility. The materials employed in NPs are assorted, including lipids, metal, silicon and silica, polymers, proteins and carbon [3,4].

Specifically, NPs possess physical and chemical properties that differ from those of bulk materials [1,5]. In addition, the flexibility of nanotechnology allows the development of safer, yet more effective, diagnostic, therapeutic, and imaging modalities [2,5].

Among several applications of nanomaterials, the use of NPs in dentistry constitutes a novel field, where a few research studies have been carried out. The most relevant pathologies in dentistry are caries, periodontal disease, and endodontic infections [6,7,8,9]. Endodontics is the field of dentistry that treats the disorders of the dental pulp and its consequences. According to the American Association of Endodontics, 15 million root canal treatments are performed annually in the USA. In this area, the World Health Organization [1] estimates that in industrialized countries, oral diseases constitute the fourth most expensive pathologies, expressed in direct cost [10]. Many oral dental pathologies are highly difficult to treat and prevent due to the restrictive dental barriers and the complex anatomy of the root canal system, which does not allow active compounds to arrive at the target site [9]. Specifically regarding to endodontic disinfection, biofilms, as well as the complex anatomy of the root canals, allows endodontic pathogens to be hidden in areas that are inaccessible for irrigating preparations [11]. Therefore, effective disinfection of the root canal system still constitutes one of the hallmarks for successful endodontic treatment [12]. This therapy is carried out using mechanical instrumentation to achieve effective microbial reduction before filling the root canal with an inert filling material. However, a major challenge in root canal treatment is the inability of the current cleaning procedures to eliminate bacterial biofilms surviving within the anatomic complexities of the root canal system [13]. Despite efforts to develop new irrigation instruments, the rate of treatment failure has not decreased below 18–26% during the past few decades. To solve these problems, NPs could offer a solution being able to transport the active compounds and deliver them effectively in a sustained manner [14,15]. Moreover, some NPs have also shown intrinsic antibacterial potential such as silver or chitosan NPs and they have also proven to be much more efficient, with good interaction properties and suitable surface chemistry compared to conventional materials [11].

In this review, NPs and their specific uses in dentistry will be discussed. In addition, an overview of dental anatomy and drug delivery barriers will be explored. The types of NPs and their advantages and disadvantages will be reviewed for endodontic purposes, with special emphasis on biodegradable NPs. In this area, NPs will provide a new paradigm shift in dentistry. This review will highlight the latest studies developed using nanotechnological tools for endodontic disinfection, their mechanisms of action and the future trends in nanodentistry aimed towards endodontics disinfections.

## 2. The Oral Cavity and the Dentin-Pulp Complex

**Figure 1:**
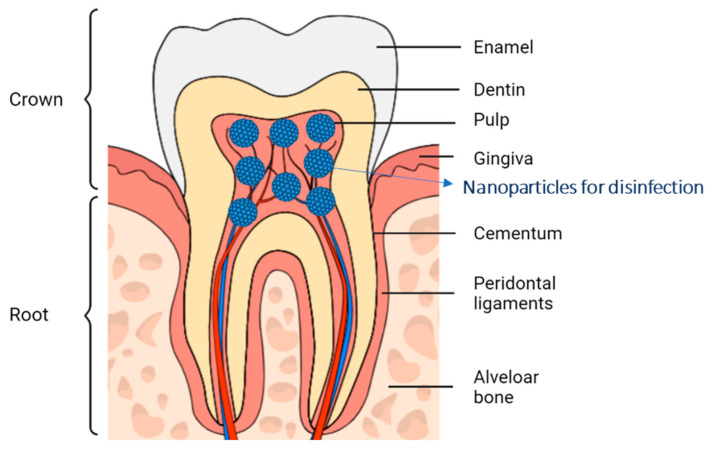
Tooth anatomy divided in crown and root and nanoparticles localization for endodontic disinfection [24,25].

The oral cavity is the most anterior subdivision of the digestive tract [16]. It is formed by the following structures (Figure 1): lips, oral mucosa, teeth, the hard palate, floor of mouth, labial frenulum, upper and lower gum, and the anterior two thirds of the oral tongue [17,18]. Despite their proximity, each area has different structures and anatomical characteristics, to provide different functions. This leads to differences in barrier properties and permeabilities that have to be taken into account to develop suitable and effective drug administration [18].

### 2.1. Anatomical Structures of the Dentin-Pulp Complex

The teeth are constituted by crown and roots. In addition, related to their tissues, teeth can be classified into two main groups: mineralized hard tissues (enamel, dentin, and cementum) and non-mineralized soft tissues (pulp) [19]. Enamel is the outer aspect of the crown and is also the most mineralized substance in the human body, composed mainly of hydroxyapatite, a crystalline calcium phosphate [20]. The next layer is the dentin which constitutes a major part of the crown and roots and is more porous than the enamel. It is composed of microtubules that connect the dental pulp and the root surface. The distribution of microtubules ranges from 15,000 × mm^2^ in the outermost zone to 45,000 × mm^2^ in the zone closest to the pulp. Inside the pulp, there is pulp tissue and odontoblast. Tubules of teeth branch into nano-tubuli, which could be observed on perpendicularly cut sections [21]. Dentin tubules are hollow microscopic channels that travel from the pulp through the dentin, ending right beneath the enamel or cementum. Pulp is vascularised by blood vessels or nerves, in contrast to enamel that does not present the mentioned structures [22].

The dentin surrounds the pulpal tissues within the tooth. In the radicular portion, dentin is surrounded by root cementum. Cementum is the tissue layer that covers the roots of a tooth and surrounds the underlying dentin which has direct contact with the periodontal ligament [23].

The morphology of the roots differs depending on each tooth type, since they can either be single rooted or multirooted. Additionally, from top to bottom, root structures are divided into coronal (which correspond to the crown of the tooth) and cervical (located in the amelo-cemental junction) (Figure 1) [19]. In the inner part of the teeth, the pulp runs inside the root canals, which present great morphological variability with multiple anatomical varieties, which makes their preparation and debridement difficult from a chemical-mechanical point of view. The pulp is a neuro-vascular complex that supplies nutrients, provides sensory functions and controls blood flow [9].

The main function of the dentinal tubules is to transport nutrients and fluids, hydrating the tooth and serving as a transducer of physical signals to sensory responses. These channels act as blood vessels in the hardest layers of the teeth, from the pulp chamber to the dentin layer [24]. These network of canals extend radially from the pulp (inside the tooth) towards the enamel-dentin junction and the cementum (outer areas).

### 2.2. Bacterial Infections of the Pulp and Dentinal-Pulp Complex

In the oral cavity, there are distinct microenvironments with several microbial communities that grow on the teeth and the epithelial surfaces [26]. In this microenvironment, around 50 different genera of bacteria can be found such as Firmicutes, Proteobacteria, Actinobacteria, and Bacteroidetes [27]. Usually, host and external microbial communities maintain a homeostatic balance, contributing to the oral health cavity by excluding harmful pathogens [26]. Therefore, in the oral cavity, the local immune system strikes a delicate balance to perform an effective immune surveillance without exuberant inflammatory responses, tolerating commensals and innocuous antigens [27].

Despite this, bacterial infections are usually represented as the most important aetiological factors in pulpal and periapical diseases. To prevent these infections, the pulp is surrounded by a strong mechanical support which protects it from the microbial oral environment. This is formed by enamel, cementum and dentine [28]. Nevertheless, the pulp could lose these protections trough diverse external factors such as caries, cracks, fractures and open restoration margins. This can cause the oral microbiota or their toxins to enter in the pulp, leading to inflammation that can end up in necrosis of the pulp tissue [29]. The pulp space is located in a complex system of root canals with a high anatomical variety that includes isthmuses, lateral canals, accessory canals, dilacerations, apical deltas and other abnormalities, which make their mechanical preparation and disinfection extremely difficult. Moreover, infections produced in the dentino-pulpar complex can lead to serious pathologies such as cellulitis, apical abscess or general disorders [28]. If the infection spreads from the maxillary teeth, it could cause other pathologies such as purulent sinusitis, meningitis, brain abscess, orbital cellulitis and cavernous sinus thrombosis. On the other hand, infections from the mandibular teeth may cause Ludwig’s angina, pericarditis or emphysema, among others. Furthermore, it should also be taken into account the emotional damage and nutritional problems that can be caused by the extraction of infected teeth [30].

### 2.3. Current Bacterial Disinfection Techniques

The current cleaning and shaping of root canals is based on the use of chemomechanical debridement to achieve optimal bacterial disinfection [31]. Among the most commonly used current irrigants are sodium hypochlorite and chlorhexidine [32]. Sodium hypochlorite is the irrigant most widely used in endodontics due to its ability to solve organic and inorganic tissues [33]. However, its main drawback is the adverse effects that can be produced due to its extrusion trough the apical foramen. Furthermore, the literature shows a low potency applied in vivo which does not correspond to the excellent results obtained in vitro. These discrepancies may be due to sodium hypochlorite, low penetration on the dentinal tubules and the buffer effect produced on the dentin [34]. Moreover, other methods used to disinfect the radicular conducts are Ethylenediaminetetraacetic acid (EDTA), Qmix^®^, MTAD^®^, Iodine Potassium Iodine (IPI) and Hydrogen peroxide [35,36].

Despite all these advances, current scientific evidence shows that the success rate of disinfection therapies in endodontics is around 85% [37]. Taking these data into account, the use of intra-canal medication to achieve greater bacterial disinfection has been extensively studied. Among all, the most commonly used medication is calcium hydroxide [13].

Calcium hydroxide constitutes the first choice for antibacterial disinfection in cases of teeth with infected root canals [38,39,40]. Its antimicrobial property is due to the ionic dissociation of calcium hydroxide upon contact with aqueous fluids, dissociating into calcium and hydroxyl ions [40]. Hydroxyl ions are highly oxidising free radicals with a high reactivity that can cause damage to the bacterial cytoplasmic membrane; denaturation of key proteins and enzymes and/or DNA damage. On the other hand, calcium ions released from calcium hydroxide produce a stimulation of the synthesis of fibronectin by the cells of the dental pulp that may cause dental pulp cells to differentiate into mineralised tissue-forming cells [41].

However, one of the reported shortcomings of calcium hydroxide is the deactivation of its active antibacterial capacity by contacting with dentinal tissue inside the root canal. This fact may be due to the inhibitory effect of dentine, as is has been previously demonstrated for various irrigants and root canal medicaments [39]. This inhibitory impact of dentin on the antibacterial activity of calcium hydroxides may be related to dentin buffering action against the primary cause of its influence, the hydroxyl ion, lowering the antibacterial potential of calcium hydroxide [42].

## 3. Nanoparticles in the Medical Field

NPs have shown a wide variety of applications, especially in the medical field. These applications range from in vitro diagnostic assays to in vivo localised imaging, drug delivery, treatment and the prevention of several diseases [43]. In addition, NPs constitute a suitable vehicle against bacterial infections due to their ultra-small size, large surface-area-to-mass ratio, increased chemical reactivity, high stability and thermal conductivity [44,45,46]. Moreover, antimicrobial effects are derived from the large surface area and high charge density of NPs which interact with the negatively charged surface of bacterial cells, providing enhanced antimicrobial activity of the encapsulated compound.

### 3.1. Nanoparticles in Endodontics

Nanostructured systems have demonstrated important applications in the medical field, especially in dentistry, coining a novel research field named nanodentistry [47,48]. These systems have been used in dentistry to polish the enamel surface, teeth whitening, prevention of caries, as a desensitizing agent or dental filling, among others [49]. Moreover, among several dental diseases, endodontics disinfection constitutes an unmet medical need that may be overcome using NP [50].

According to the available data, there are several types of NPs used in endodontics. Among them, biodegradable (e.g., polylactic acid), inorganic (e.g., silica) and metal (e.g., gold) NPs are the most commonly employed [50]. Therefore, the medical applications of these NPs will be discussed in the following subsections [51].

### 3.2. Biodegradable Nanoparticles

Biodegradable NPs constitute promising carriers for the administration of a large variety of drugs, being able to provide the targeted delivery of drugs, improved bioavailability, prolonged therapeutic efficacy, diminished drug resistance and reduced side effects [52]. Moreover, they are one of the most widely used for drug delivery [53].

Among several biodegradable NPs, polymeric NPs are the most widely used. They are made of polymers that can be synthetic or natural. Synthetic polymers are based on polyesters such as polylactic acid (PLA), poly(lactic-co-glycolic acid) (PLGA), poly (ε-caprolactone) (PCL) or poly-β-hydroxybutyric acid (PHB) [52,54,55]. Natural polymers include chitosan, cellulose, gelatine, gliadin and different polysaccharides such as pullulan.

Polyester-based NPs are one of the few synthetic polymers that have entered clinical trials in drug delivery application due to its excellent safety profile [56]. PLA, PLGA, PCL and PHB are not only degradable in the physiological environment, they are also bioresorbable, thus indicating that their degradation product or intermediates can be eliminated through natural pathways by simple filtration or metabolism [57]. Degradation occurs by several mechanisms, but the main one is through hydrolysis of the ester bonds. Moreover, NPs fragments can also be taken up by macrophages phagocytosis. On the other hand, PLA and PCL may also enter the citric acid cycle and be eliminated [58]. In addition, PLGA is authorized by the Food and Drug Administration (FDA) and it is used as a carrier for controlled drug release [59].

In this area, polyester derivatives have been approved in combination with different drugs. Additionally, a PLA micelle containing paclitaxel (Genexol-PM) has been approved in South Korea, India, and Indonesia for the treatment of breast, ovarian and lung cancer; it is now conditionally approved by the FDA pending a full demonstration of effectiveness in the EU [60]. There are also chemotherapeutic formulations of PLGA that are enriched by its different properties (shape, support or size) currently approved by the FDA and available on the market for various types of cancer treatment. The most prominent among them are based on PLGA microspheres such as Lupron Depot^®^ (Abbvie Endocrine Inc.) or Eligard^®^ (Tomar Therap), the latter being a PLGA-based gel [61]. Moreover, several studies using PLGA nanoparticles in the medical field have been carried out. In this area, research using PLGA NPs has proven a utility-wide variety of diseases such as ocular, brain or dermal pathologies [54,62,63,64,65].

Natural polymers are extracted from animals, plants, bacteria and fungi. They can be divided into two main groups: polysaccharides and protein-based polymers. As a result, drug delivery with high loading efficiency and minimally invasive behaviour can be achieved. Natural polymers are usually derived from natural sources, such as chitosan from chitin, and for this reason they generally have good biocompatibility and biodegradability [66]. The use of polysaccharides and polymers for drug delivery has shown promising results, especially in cancer therapy [67,68,69]. This type of encapsulation has given good results with chemotherapeutic agents of plant origin, such as paclitaxel, which showed suitable controlled release behaviour and greater antitumoral efficacy in MDA-MB231 (human breast tumoral cells) [70]. Similar results have also been observed with artificial agents, such as in the study developed by Kim et al. in which they used hydrophobic cholanic acid-modified glycol chitosan (HGC) to encapsulate cisplatin (CDDP), which proved to be a promising vehicle for effective anticancer drug CDDP delivery [71]. Even though chitosan is approved in dietary use, wound dressing applications and cartilage formulations, there is not yet a chitosan NPs-based formulation approved for medical purposes [72].

### 3.3. Inorganic Nanoparticles

The main inorganic materials used to produce NPs are silicon, graphene and silica [73]. These NPs offer a wide variety of sizes, structures and geometries according to their production methods [46]. Inorganic materials have a high chemical, thermal and mechanical resistance, which facilitates their production and chemical modification. However, this higher strength may also hinder their penetration into tissues due to their lack of flexibility. In addition, they offer good biocompatibility, low immunogenicity, easy scalability, cheap production and high drug loading capacity due to their porosity. The latter constitutes a crucial property since inorganic NPs can be modified to possess pores with different diameters. This allows the transport of several active compounds, from small drugs such as chemotherapeutic agents to large proteins or oligonucleotide chains [73].

Among several materials, silica NPs are one of the most commonly used inorganic nanomaterials. They can degrade into silicic acid or small silica species in certain aqueous media and offer suitable biocompatibility [74]. The most commonly used applications of silica NPs are diagnostic imaging [75,76] and drug delivery [77,78]. In fact, the FDA has approved fluorescent silica nanoparticles for human clinical trials named Cornell dots. Cornell dots range in size from 3 to 6 nm and are made of iodine 124, its primary target for positron emission tomography (PET) imaging, and cyclo-(Arg-Gly-Asp-Tyr) peptides (cRGDY) for molecular orientation. Cornell dots can be modified with radioisotopes or contain an NIR dye (i.e., Cy5), whereby they could be used as hybrid PET optical imaging agents [79]. These showed a significantly improved target-background ratio and higher sensitivity, showing great potential for cancer diagnostics [80]. Other common inorganic NPs are those made from calcium phosphate. These have been used successfully for gene and drug delivery [81]. A summary of inorganic nanoparticles for medical purposes can be found in Table 1.

### 3.4. Metal Nanoparticles

Metal NPs have unique physical, electrical, magnetic and optical properties [82]. They are of particular interest for applications such as diagnostics, imaging and photothermal therapies due to their magnetic, radioactive and plasmonic properties. However, the main drawback for their clinical application is limited due to the low solubility and toxicity problems at both human and environmental levels [83].

The main materials used for the production of metal NPs are gold, iron and silver. The most widely studied are Gold NPs (AuNPs) due to their photothermal properties, and are used in various forms such as nanospheres, nanorods, nanostars, nanoshells and nanocages [46]. AuNPs possess free electrons on their surface that oscillate continuously at a frequency that depends on their size and shape. In addition, they can be easily functionalized [84]. Moreover, some of them are commercialized, as is the case of gold nanocapsules called AuroLase^®^ used in the treatment of brain and neck tumours [85].

In addition, iron oxide is another frequently investigated material for the synthesis of inorganic NPs and constitutes the majority of FDA-approved inorganic nanomedicines. Iron oxide NPs are composed of magnetite (Fe_3_O_4_) or maghemite (Fe_2_O_3_), which possess superparamagnetic properties at certain sizes. This allows them to have properties that make them suitable contrast agents, drug delivery vehicles and thermal-based therapeutics [46]. Moreover, Fe_3_O_4_NPs also exhibit good biocompatibility, which has led to the clinical approval of several drugs based on iron oxide NPs [74]. This is the case of Feridex^®^, an MRI contrast agent [86]. Moreover, Fe_3_O_4_NPs are also used in cancer treatment, as is the case of NanoTherm^®^. NanoTherm^®^ was the first therapeutic NP formulation approved in Europe (2010). It is effective against brain tumours and it is based on magnetic NPs with an iron oxide core that by applying an alternating magnetic field, causing rapid rotation of NPs that induces heat in the tumour by friction, causing cancer cells to be irreversibly damaged or sensitized to receive additional chemotherapy or radiotherapy [87,88].

Furthermore, silver NPs (AgNPs) also constitute a highly relevant group of metal NPs. AgNPs stand out in particular for their antibacterial role. AgNPs possess chemical stability, high electrical and thermal conductivity and catalytic activity [89]. AgNPs have been applied in different fields, such as textiles, cosmetics, food industry and biomedicine. In the biomedical field they are gaining strength, especially for their applications as antimicrobial agents, in molecular diagnostics, and as carriers of chemotherapeutics [90]. AgNPs have been used as disinfectants and are effective against several bacterial strains such as *Escherichia coli* and *Staphylococcus aureus* [91,92,93], *Mycobacterium tuberculosis* and *Chlamydia trachomatis* [94], among others. Furthermore, these NPs have also been used in combination with antibiotics such as cefazolin (CEF), mupirocin (MUP) or gentamicin (GEN), with good results against *Staphylococcus aureus*, *Pseudomonas aeruginosa* and *Escherichia coli* [95]. In addition to their antimicrobial and antifungal applications, the characteristic conductive properties of AgNPs allows them to be employed for photothermal, laser, and radiation therapies in order to enhance anti-tumoral therapy. For this reason, they have been extensively studied against different tumours (leukaemia, breast cancer, hepatocellular carcinoma, lung carcinoma and colon carcinoma) [96,97]. Moreover, a summary of the medical applications of metal NPs can be found in Table 2.

### 3.5. Mesoporous Calcium Silicate

Calcium silicate-based materials play an important role in the development of endodontic materials that induce bone/cementum tissue regeneration and inhibit bacterial viability [98]. In this sense, mesoporous calcium silicate NPs combines the properties of calcium silicate but also possesses pores that have diameters between 2 and 50 nm that can be used for drug delivery. In this sense, Huang et al. loaded some antibiotics such as gentamicin in order to potentiate its antibacterial properties [98].

Furthermore, these NPs are also useful in filling the apical third of the root canals due to their property of being highly viscous in nature [11].

### 3.6. Nanoparticles Functionalization

Despite the relevant effects and beneficial features of NPs, the number of nanomedicines available in the market is far below the projections expected for the field [83]. To solve these complications, there are new mechanisms to vectorize NPs to a specific site of action by adding specific compounds to the NPs surface, allowing them to be directed towards specific tissues [99]. Functionalization can be achieved using several compounds such as peptides, antibodies, stabilizers, or imaging agents [100]. Although in endodontics limited examples of functionalized NPs can be found, in other areas, several studies have been developed using targeted NPs.

Functionalization can be achieved my modifying the surface of the NPs, as in the study carried out by Folle et al. The authors developed a thymol-loaded surface-functionalized PLGA NPs for topical administration that enhanced the anti-inflammatory, antioxidant, and anti-acne healing activities of thymol. In this study, a synergistic activity between TH-NPs and their surface functionalization using chitosan for their action against acne was demonstrated [101]. Furthermore, together with the increase of viscosity, the addition of PEG to the nanoparticles’ surface is a recognised strategy to reduce nanoparticles clearance [56,62,102,103].

Moreover, one of the most novel functionalization mechanisms are those carried out with antibodies. This is the case of the study carried out by Marega et al. using plasma-polymerized allylamine-coated AuNPs, bioconjugated with a monoclonal antibody targeting epidermal growth factor receptor. With these, it was possible to overexpress the epidermal growth factor receptor, as determined by ELISA and Western blot assays. In addition, in vivo targeting of these receptors was also observed [104]. In this area, functionalization using customized peptides has also shown great potential. Several studies have been carried out using cell penetrating peptides aimed to increase the passage of biodegradable NPs trough cellular membranes conferring increased selectivity to the NPs. Specifically, in the studies carried out by Gonzalez-Pizarro et al., they developed PLGA NPs encapsulating fluorometholone for ocular drug delivery [105]. Increased ocular anti-inflammatory effect was demonstrated. Similarly, other researchers also encapsulated drugs into PLGA-peptide targeted nanocarriers, proving an increased passage towards cell membranes such as cornea [106,107], brain [108], colon [109] or tumoral cells [109].

Furthermore, a different strategy to increase the remaining time of NPs in the tissues is their dispersion into gelling systems [110]. Among all, stimuli-forming gels constitute one of the most novel advances. In this area, Esteruelas et al. encapsulated Riluzole into PLGA NPs and these were dispersed in an in situ gelation system to improve the biopharmaceutical profile of Riluzole after ocular administration. As a result, the gel formulation increased the contact of the NPs with the ocular surface and demonstrated the ability to be distributed in the posterior segment of the eye for 24 h after application [55].

## 4. Biodegradable Nanoparticle in Endodontics Disinfection

The most commonly used antimicrobials for effective endodontic microbial reduction are usually intracanal medication and irrigants combined with mechanical instrumentation. Despite advances in this method, the treatment failure rate comprises between 5–25%, depending on pulpal and periapical status [111]. This fact makes it clear that there is an urgent medical need for novel disinfectant strategies in endodontics [13].

### 4.1. Dentinal Biofilms

Bacterial biofilms are considered the main cause of root canal infection. Oral biofilms are a structured bacterial community with a wide range of microbes embedded in a self-made matrix of extracellular polysaccharides (EPS). This biofilm is a virulence factor for many oral infectious diseases, such as dental caries, gingivitis, periodontitis, periapical periodontitis and peri-implantitis [112]. The life cycle of biofilms consists of an initial bacterial adhesion that may be a reversible attachment, subsequent colonization (irreversible), growth, maturation characterized by the enhanced production of EPS and, finally, its dispersion in the environment [113,114].

The search for biofilm eradication compounds is influenced by the complexity of the oral cavity and the rapid clearance of saliva, due to the fact that topically applied antibacterial agents are not retained in sufficient concentrations, or for long enough to perform their action [115]. In this area, novel advanced disinfection strategies involve antibiofilm treatment in the root canal using antibacterial NPs, which are showing significant antimicrobial potential [13].

**Figure 2:**
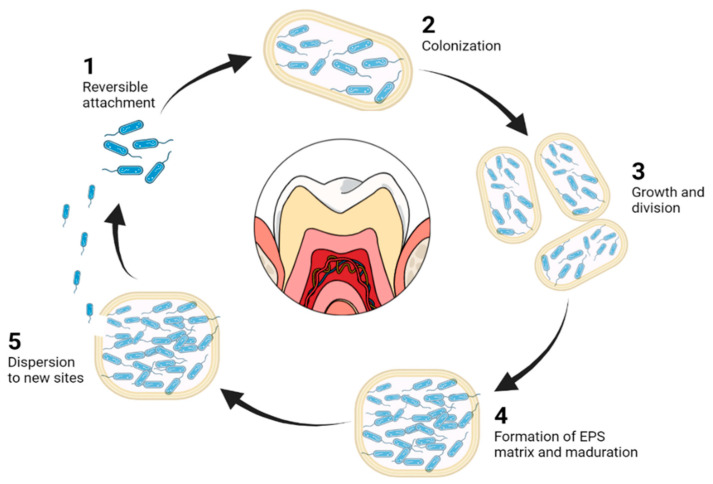
Stages in biofilm formation. (1) Reversible adhesion of bacteria to the infection surface by means of their superficial appendages (pili and flagella). (2) Irreversible adhesion, forming microcolonies. (3) Growth and cell division. (4) Bacterial secretion from the extracellular polymeric matrix, composed of proteins and polysaccharides and biofilm maturation. (5) Bacterial detachment and migration to different environments. Based on [113].

Although they are not biodegradable, metal NPs are the most widely used in endodontics disinfection. Among them, one of the components with the most promising potential against biofilms is Coper oxide (CuO). CuONPs have been reported as suitable alternatives to control biofilm formation within the oral cavity. The mechanism of action of CuONPs is the restriction of bacterial growth by hindering the passage inside cell membranes of the majority of bacterial strains [116]. Although CuNPs have also been used, they present rapid oxidation when exposed to air, which, added to their non-biodegradability, makes their application limited [117]. Stages of biofilm are reproduced in Figure 2.

Moreover, AgNPs also possess antibacterial properties able to penetrate the biofilms and release silver ions. In this field, a comparative study of AgNPs and free silver nitrate was carried out demonstrating that AgNPs had a higher antibacterial power than free silver ions [118]. AgNPs offer a strong broad-spectrum antibacterial agent (gram-positive and gram-negative bacteria), and also suitable stability. The antibacterial activity of the AgNPs results from the damage to the bacterial cell membrane, in addition to the interaction with the disulphide or sulfhydryl groups of the enzymes, causing the interruption of the metabolic processes [117]. However, the toxicity of AgNPs is still under evaluation [119].

### 4.2. Biodegradable Nanoparticles in Endodontics

**Figure 3:**
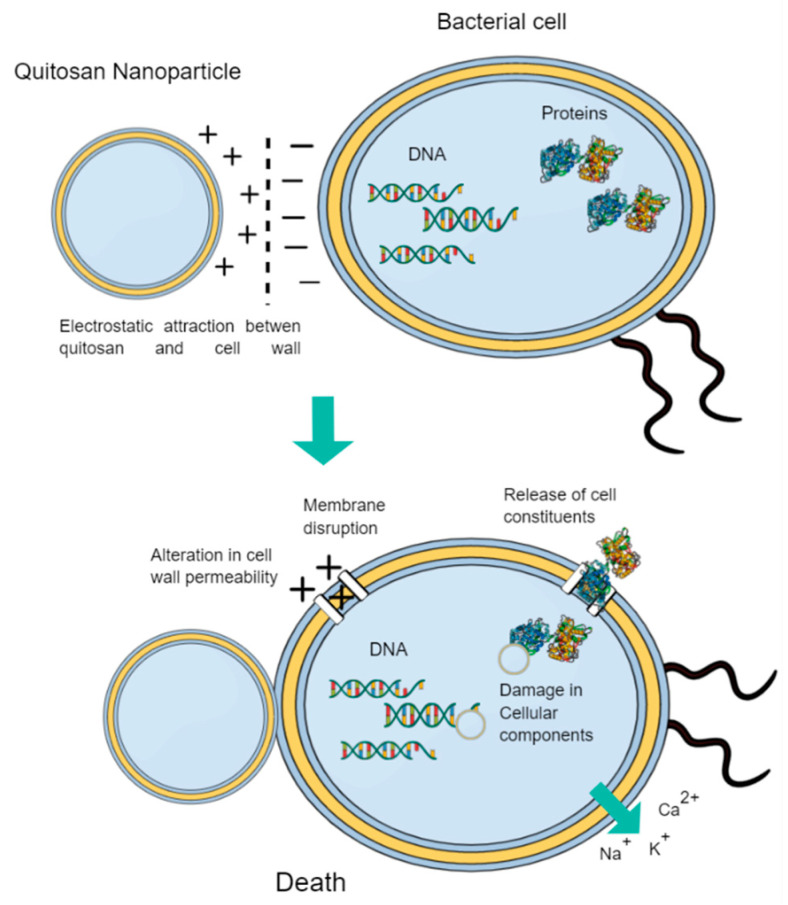
Chitosan NPs antibacterial mechanism of action. Based on [13,128].

Biodegradable NPs have drawn attention among professionals due to their suitable properties and decreased side-effects. One of the most widely used polymers in endodontics is chitosan or poly [1,4-b-D-glucopyranosamine]. Chitosan is a deacetylated derivative of chitin and its structure is similar to the components of the extracellular matrix, being able to reinforce collagen constructions [13]. Chitosan is a versatile compound in terms of its forms and functions, as demonstrated by its excellent antibacterial, antiviral and antifungal properties, which has aroused great interest in biomedicine [120]. This polymer can be loaded with different active compounds such as chlorhexidine (CHX), which has produced great results in the elimination of bacteria and biofilms in specific oral diseases [121]. Chitosan has a positive charge, which could lead to the electrostatic attraction of chitosan with negatively charged bacterial cell membranes (Figure 3). This attraction would lead to an alteration in cell wall permeability, resulting in cell rupture, leakage of intracellular proteins and components, and ultimately death of the microbial species [122]. In this area, chitosan nanoparticles (CS-NPs) have demonstrated their potential to be administered in the dentinal tubules of an infected root canal to improve root canal disinfection, overcoming its anatomical complexities. Moreover, it was observed that the effect of CS-NPs did not decrease when used together with inhibitors of the efflux pump, a mechanism of biofilm resistance to antimicrobial agents [123].

The efficacy of CS-NPs in improving root canal disinfection has been evaluated in dentin infected teeth and treated with *Enterococcus faecalis*. In this study, it was observed that dentin treated with CS-NPs resulted in significantly less adherence of *Enterococcus faecalis* than untreated dentin and a significant penetration of antibacterial NPs into the dentinal tubules was observed [124]. In addition, the capability of CS-NPs avoiding the formation of biofilms have been assessed. *Streptococcus oralis*, *Prevotella intermedia*, and *Actinomyces naeslundii* biofilms were used to infect dentin sections and the application of CS-NPs significantly reduced the antibiofilm activity. Moreover, using confocal laser scanning microscopy (CLSM), the greater penetration of the CS-NPs was also proven [13]. In another study, Carpio-Perochena et al. also evaluated the antibiofilm effect of CS-NPs through bovine dentin sections and it was observed that CS-NPs-treated sections had half of the dentin smear and bacterial recolonization inhibited [125]. Moreover, Soto Barreras et al. loaded CS-NPs with CHX to eliminate *E. faecalis*. A comparative in vitro study was carried out with chlorhexidine (CHX) and CHX loaded CS-NPs, showing a significantly greater reduction in colony-forming units on agar plates caused by CHX loaded CS-NPs [126]. Furthermore, Li et al. found that using cross-linked CS-NPs could decrease root stress distribution and improve resistance to fatigue loads in endodontically treated teeth [127]. All these studies confirm that CS-NPs present a significant potential in the disinfection of the root canal. However, there are still different barriers that must be overcome, such as the treatment time required to achieve effective bacterial elimination using these nanosystems [13].

Other biodegradable polymers widely used in endodontics are biodegradable polyesters [129,130]. These polymers (PLA, PLGA, PCL and PHB) are bioabsorbable and their degradation products can be eliminated through natural pathways [58]. These are used as carriers for antibacterial, antibiotics or different types of medicines. However, they do not possess antibacterial properties by themselves.

The mechanism of action for the elimination of microorganisms is mainly by fusion with the microbial cell membrane and the subsequent release of the antimicrobial agent [131]. In this area, our group has recently developed PLGA NPs loading Ca(OH)_2_, an antibacterial compound, obtaining a prolonged Ca(OH)_2_ release able to be distributed along the root canals [45]. Additionally, the NPs displayed a superior ability to penetrate inside the dentinal tubules compared to marketed Ca(OH)_2_. Moreover, Wang et al. also developed PLGA NPs loaded with Ca(OH)_2_, which, after their application in the dentin infected models, were able to produce a decrease in the bacterial load and their by-products [132]. Furthermore, triclosan, an antimicrobial agent with high efficacy against plaque-forming bacteria, have been loaded into PLGA and PLA NPs [133]. In this case, triclosan loaded NPs efficacy for the treatment of periodontal disease was assessed. Both formulations resulted in significant penetration into dentinal tubules, being superior in the case of PLGA NPs. In addition, a rapid release of triclosan from NPs attributed to the NPs large surface area was observed, along with a decrease in gingival inflammation [134].

Other studies have also shown the potential of PLGA to efficiently deliver active compounds within the structure of the dental tubules at a sufficient depth to exert their action [135]. Recently, other authors have carried out NPs functionalization to be used for disinfection in endodontics through photo-dynamic therapy (PDT) [136]. PDT is a novel interesting approach that could be used by loading photosynthesizers inside NPs. In this area, Pagonis et al. have also assessed PLGA NPs potential on extracted human teeth, observing positive results against E. faecalis using the photosensitizer methylene blue [137]. Therefore, methylene blue loaded PLGA NPs demonstrated the potential to be used as carriers for PDT in endodontics. A summary of biodegradable nanoparticles used in endodontics can be found in Table 3.

## 5. Conclusions

Nanodentistry constitutes one of the most novel applications of nanotechnology. In this area, NPs focused on dentistry and, more specifically, in dental disinfections, constitute an important area that still needs to be explored. Nanomaterials offer a platform towards the specific delivery of active compounds, being able to penetrate trough the root canals and fight against biofilm resistance. Specifically, biodegradable NPs have demonstrated biocompatibility and reduced toxicity. Among them, chitosan and PLGA NPs are the most widely used for these purposes, demonstrating their efficacy towards dental infections. However, only a reduced number of studies have been published in this area and, therefore, further investigations are necessary in order to develop efficient nanomedicines to avoid the high risk of failure associated with oral disinfection.

## 6. Future Perspectives

Nowadays, novel therapies in order to increase the success rates of endodontic disinfection constitute an unmet medical need. Among them, NPs are advocated as an innovative and effective tool in order to overcome the complicated dental tubules anatomy and deliver active compounds to the target site. Among several NPs types, metal NPs, especially silver NPs, are one of the most currently studied due to their suitable antibacterial effects. However, they pose serious concerns due to their toxic effects on human health and towards the environment. Therefore, efforts are being directed either to decrease the toxicity of silver NPs using functionalization or coating strategies, or towards the study and development of biodegradable NPs, such as chitosan-based NPs that possess a positive surface charge and intrinsic antibacterial capacity. Furthermore, lipid NPs also offer an alternative that might be worth exploring in order to deliver active compounds in a directed manner, using natural compounds that are safe for human use.

## Figures and Tables

**Table 1:** Summary of recent advances and types of inorganic NPs in the medical field.

Nanoparticle Material	Main Properties	In Vitro Studies	Ex Vivo Studies	References
Silica	Most commonly used inorganic materials. Great biocompatibility. Their main applications are diagnostic imaging and drug delivery.	Diagnostic imaging: In Vivo Photoacoustic Imaging of Livers Using Biodegradable Hyaluronic Acid- Conjugated Silica Nanoparticles.	Diagnostic imaging: Photoluminescent and biodegradable porous silicon nanoparticles for biomedical imaging.	[75,76]
			Drug delivery: Controllable drug release and simultaneously carrier decomposition of SiO_2_-drug composite nanoparticles.	[57]
			Double loaded self-decomposable SiO_2_ nanoparticles for sustained drug release.	[58]
Silica (Cornell dots)	Fluorescent silica nanoparticles for human clinical trials approved by FDA. These can be modified with radioisotopes or optical imaging agents. Moreover, these NPs showed a significantly improved target-background ratio and higher sensitivity for cancer diagnostics.	Clinical translation of an ultrasmall inorganic optical-PET imaging nanoparticle probe.	-	[80]
Calcium carbonate	Successfully used for gene and drug delivery.	-	Calcium carbonate nanoparticles; Potential in bone and tooth disorders.	[81]

**Table 2:** Summary of recent advances and types of metal NPs in the medical field.

Active Compound	Nanoparticle Material	Main Properties	In Vitro Studies	Ex Vivo Studies	References
-	Gold	The most widely studied in various forms due to their photothermal properties and its capacity to be easily functionalized. Some of them are commercialized (AuroLase^®^, treatment of head and neck tumours).	Gold nanoshell- localized photothermal ablation of prostate tumours in a clinical pilot device study.	-	[85]
-	Iron	Possess superparamagnetic properties at certain sizes, good biocompatibility and great properties for being a contrast agent (Feridex^®^) or against cancer treatment (NanoTherm^®^).	Cancer treatment: Plasmonic photothermal therapy (PPTT) using gold nanoparticles.	Contrast agent: Fractionated Feridex and positive contrast: In vivo MR imaging of atherosclerosis.	[86,88]
-	Silver	AgNPs stand out especially for their, chemical stability, higher electrical and thermal conductivity of metals, catalytic and antibacterial activity. In the biomedical field they are gaining strength in molecular diagnostics, and as carriers of chemotherapeutics.	Antibacterial properties: Anti-inflammatory effects of silver-polyvinyl pyrrolidone (Ag-PVP) nanoparticles in mouse macrophages infected with live Chlamydia trachomatis. Antibacterial activity of silver nanoparticles (AgNPs) in Staphylococcus aureus and cytotoxicity effect in mammalian cells. substance.	Cancer treatment: Anti-leukaemia activity of PVP-coated silver nanoparticles via generation of reactive oxygen species and release of silver ions	[71,93,97]
Antibiotics	Silver	Ag NPs have also been used in combination with antibiotics such as cefazolin (CEF), mupirocin (MUP) or gentamicin (GEN) with good results against *Staphylococcus aureus*, *Pseudomonas aeruginosa* and *Escherichia coli*.	Elucidating pharmacodynamic interaction of silver nanoparticle—Topical deliverable antibiotics.		[95]

**Table 3:** Summary of biodegradable NPs used in endodontics disinfection.

Active Compound	Nanoparticle Material	Main Properties	In Vitro Studies	Ex Vivo Studies	References
-	Chitosan	Electrostatic attraction with bacterial cell membranes. Versatile compound in forms and functions. Excellent antibacterial, antiviral and antifungal properties. High biodegradability, non-toxicity. Proven antibiofilm efficacy. Hight root canal penetration.	-	Adherence of *E. faecalis* to dentin in sectioned single-rooted teeth showing bacterial death and decreased adherence.	[124]
				Multispecies biofilm infected dentin sections proved the antibiofilm activity and CLSM determined a high penetration.	[138]
				Bovine dentin sections were infected intra-orally, the treatment result in an inhibition of bacterial recolonization on root dentin.	[125]
Chlorhexidine	Chitosan	Antibacterial spectrum that includes most of the microorganisms of the oral cavity.	Collagen membrane with *E. faecalis* infection, results significant inhibition of bacterial growing.	-	[126]
	Cross-linked chitosan	Improved resistance to fatigue loads in endodontically treated teeth.		Root canal dentin sections were subjected to nanoindentations before/after treatment, showing a decrease of stress root.	[127]
Ca(OH)_2_	PLGA	Bioabsorbable by simple filtration or metabolism. Prolonged release. Hight root penetration.	-	Single-rooted human teeth were treated with PLGA NPs and observed with confocal microscope, demonstrating higher NPs penetration.	[45]
				Single-rooted teeth infected with *E. faecalis* and treated, the result was a decrease in bacterial species and their by-products.	[132]
Triclosan	PLGA and PLA	Hight root penetration. Hight encapsulation efficiency. Large surface area.	-	Beagle dogs with induced periodontitis were treated showing a decrease in gingival inflammation.	[134]
Chlorhexidine	PLGA	Potent antibacterial efficacy. Slow degradation and gradual chlorhexidine release profile. Increased NPs penetration.		Extracted teeth were connected to experimental setup simulating pulpal hydrostatic pressure, the result was a potent antibacterial efficacy, and gradual degradation pattern.	[135]
Methylene blue	PLGA	Potent antibacterial effects. Novel antimicrobial endodontic treatment.	-	*E. faecalis* infected root canals were treated and irradiated with red light at 665 nm obtaining a CFU levels significantly lower.	[139]

## Data Availability

Not applicable.
